# Pressure Ulcers—A Longstanding Problem: A 7-Year Neurorehabilitation Unit Experience of Management, Care, and Clinical Outcomes

**DOI:** 10.3390/diagnostics13203213

**Published:** 2023-10-14

**Authors:** Angelo Alito, Simona Portaro, Giulia Leonardi, Carlotta Ventimiglia, Francesco Bonanno, Domenico Fenga, Cristiano Sconza, Adriana Tisano

**Affiliations:** 1 Department of Biomedical, Dental Sciences and Morphological and Functional Images, University of Messina, 98125 Messina, Italy; 2 Physical Rehabilitation Medicine Department, University Hospital A.O.U. “G. Martino”, 98125 Messina, Italy; 3 Department of Adult and Developmental Human Pathology, University of Messina, 98125 Messina, Italy; carlottaventimiglia@gmail.com; 4 Department of Clinical and Experimental Medicine, University of Messina, 98125 Messina, Italy; atisano@unime.it (A.T.); ciccio.bonanno@yahoo.it (F.B.); 5 Department of Orthopaedics and Traumatology, University Hospital A.O.U. “G. Martino”, 98125 Messina, Italy; 6 IRCCS Humanitas Research Hospital, Rozzano, 20089 Milan, Italy; 7 Department of Biomedical Sciences, Humanitas University, Pieve Emanuele, 20090 Milan, Italy

**Keywords:** health services research, inpatient rehabilitation, multidisciplinary/interdisciplinary rehabilitation, neurologic disorders, quality improvement and patient safety, wound management

## Abstract

Background: Neurological disease patients present an increased risk of developing pressure ulcers. The primary aim of this study is to evaluate the incidence and prevalence of pressure ulcers and their impact on length of stay and functional recovery. Methods: A retrospective study was conducted in a neurorehabilitation unit over a seven-year period. Data collected include demographic data, length of stay, functional evaluation, risk of pressure ulcers development, nutritional status, and skin. Pressure ulcers were classified according to the European Pressure Ulcer Advisory Panel System. Results: Data from 816 patients were analyzed. On admission, the authors found 236 pressure ulcers in 131 patients (about 16%), divided into stage I (25%), stage II (50%), and stage III–IV (25%). The most common sites were the heel (36%) and sacrum (29%). Among the risk factors for the development of pressure ulcers, malnutrition played a significant role, with approximately 76% of patients with pressure ulcers having mild to moderate malnutrition. Conclusion: The presence of pressure ulcers seems to have a negative impact on the functional recovery of patients, as shown by the outcome scales and the average length of stay: 51 days versus 36 days (*p* < 0.01).

## 1. Introduction

A pressure ulcer (PU) is defined as a localized skin damage and/or its underlying tissues, caused by pressure and share, particularly at bony prominences [1]. PUs are an important clinical issue in hospitalized patients with reduced mobility and represent a burden for the health care system [2,3]. Several neurological diseases (i.e., spinal cord injuries (SCI), stroke) with strength, sensitivity, and/or state of consciousness alterations and/or cognitive impairments may be more vulnerable to such lesions [4,5,6]. The prevalence and incidence of PUs in hospitalized patients are discordant, ranging, respectively, from 2.7 to 29% [7], and from 3.5 to 69% [8,9]. Such discordance may depend on the health-care setting considered: in the intensive care unit (ICU), PU prevalence ranges from 3 to 20% [10], in long-term care facilities it is about 11% [11], and in home-care settings it is 9.12% [12]. Nevertheless, in patients admitted to rehabilitation units and/or long-term care units there is an incidence of 5.23% [13]. The discordance may also depend on external (i.e., pressure, friction, shear forces, and moisture) and internal factors (i.e., fever, malnutrition, anemia, and vascular dysfunctions) [14]. External factors are suitable for preventive interventions [15,16] whereas the internal ones depend on the patient’s clinical condition [17], i.e., malnutrition [18,19,20]. Low albumin and hemoglobin serum levels may represent a negative prognostic factor [21,22]. The most common PU classification is the international National Pressure Ulcer Advisory Panel/European Pressure Ulcer Advisory Panel (NPUAP/EPUAP) [23], which consider five (1–4 + unstageable) PU stages. An improper PU management may interfere with rehabilitation and functional recovery [24], worsening patients’ quality of life [25,26], prolonging the time of hospitalization [27], and sometimes leading to surgical treatments [28]. Thus, an accurate PU staging is important to correctly handle and monitor ulcer evolution [29]. Despite the interest in PUs, few studies have addressed this issue in an inpatient neurological rehabilitation setting, so the aim of this study is to assess the incidence and prevalence of PUs and their impact on length of stay (LOS) and functional recovery in a neurorehabilitation unit. 

## 2. Methods 

### 2.1. Study Setting and Design

The authors conducted an observational, retrospective, monocentric study. Patients were selected from the medical records of patients consecutively admitted to the Neurorehabilitation Unit of the University Hospital “G. Martino” of Messina, from 2002 to 2007. The study was reported according to the STROBE guidelines (Strengthening the reporting of observational studies in epidemiology) [30]. The Internal Review Board of the Neurorehabilitation Department approved the study and the medical record consultation. Considering the study’s retrospective design, an ethical review and approval code from the Ethics Committee were not required in accordance with institutional requirements. On admission, all patients signed an informed consent form to be included in scientific studies. Informed consent was signed by parents or legal guardians in the case of minors.

### 2.2. Study Population

Medical records were retrospective analyzed and data were collected and analyzed by the authors, with full confidentiality and anonymity guaranteed. All patients were hemodynamically stable. The inclusion criterion was admission to the Neurorehabilitation Unit of the University Hospital “G. Martino” in that period. The exclusion criteria were absence of a legal representative to sign the consent form and a duration of hospitalization of less than one week.

### 2.3. Demographical and Clinical Data

On admission, clinicians collected the following data: demographic characteristics, department of origin, main diagnosis, nutritional status (albumin, hemoglobin), functional evaluation scales, PU stage, PU risk assessment scales (i.e., Norton Scale, Braden Scale, Waterlow Scale). 

PU classification, according to the EPUAP/NPUAP staging system, was used as follows. Stage I: undamaged skin with hyperemia that does not disappear after pressure; stage II: partial-thickness skin lesion affecting the epidermis and/or dermis with a superficial viable wound manifesting as an abrasion or a serum blister; stage III: full-thickness skin lesion with subcutaneous tissue degeneration or necrosis and possible extension not beyond the muscle fascia; stage IV: massive degeneration, tissue necrosis or muscle, bone or supporting structure exposition and damage; unstageable: full-thickness lesion covered by slough or eschar (E) such that it does not allow grading [23].

The Norton Scale [31] assesses five parameters (score 1–4), including physical and mental status, incontinence, mobility, and ambulation. The sum establishes the risk index: <10 very high risk; 10–13 high risk; 14–18 medium risk; >18 low risk. The Braden Scale [32] is divided into 6 items evaluating sensory perception (1–4), skin wetness (1–4), physical activity (1–4), mobility (1–4), nutrition (1–4), and friction and shear (1–3). The sum defines the risk class: ≤9 severe risk, 10–12 high risk, 13–14 moderate risk, ≥15 low risk. The Waterlow Score evaluates body mass index (BMI), skin and nutritional status, sex, age, continence, mobility, and special risk factors such as malnutrition, neurological deficits, recent trauma or major surgery [33]. The total score assigns the risk class: ≥10 at risk, ≥15 high risk, ≥20 very high risk. In SCI patients, the Spinal Cord Injury Pressure Ulcer Scale (SCIPUS) was used, evaluating 15 items (score 0–25); the higher the score, the higher the risk of developing PUs (cut off > 6 indicates patient at risk) [34]. 

The Pressure Ulcer Scale for Healing (PUSH) [18,35] was used to evaluate PU evolution in all stages apart from Stage I, which was evaluated only through clinical observation. Specifically, the PUSH scale considers the skin surface, exudation, and type of tissual injury (score 0–17), with a score inversely proportional to PU improvement. In patients with PUs and no PUs but with a medium/high score in the risk development scales, some preventive measures were used, such as an anti-bedsore mattress, an electric articulating bed, a two-hour postural change program, and positioning aids. All patients underwent a daily 180-min rehabilitation program including physiotherapy, speech therapy, and occupational therapy. PU management depended on the PU stage including topical care, avoiding pressure, shear, and friction, and maintaining skin hydration (stage I–II), and, if necessary, surgical approaches (stage III–IV + E) [36]. A serum albumin level ≤ 3.4 mg/dL was considered as a risk factor for the occurrence of pressure injuries classifying malnutritional state into mild (albuminemia 2.8–3.4 g/dL), moderate (albuminemia 2.1–2.7 g/dL), and severe (albuminemia < 2.1 g/dL) [37,38]. 

### 2.4. Functional Assessment

All patients underwent a disability assessment on admission and at discharge. While in hospital, all patients received a comprehensive multidisciplinary rehabilitation program tailored to their clinical needs. Functional assessment was performed using the Functional Independence Measure (FIM) [39] and Barthel Index (BI) [40], also analyzing their efficiency and effectiveness. Efficiency is considered as the average daily scale increment during hospitalization in a rehabilitation unit [41,42], calculated according to the following formula:

$$Efficiency=\frac{\left(discharge\;score-admission\;score\right)}{days\;of\;hospitalization}$$

The potential score increase is lower in patients who started from higher scores, so the achievement of a potential improvement can be measured through effectiveness [41,42] with the following formula:

$$Effectiveness=\frac{\left(discharge\;score-admission\;score\right)}{\left(maximum\;score-admission\;score\right)}\times 100$$

### 2.5. Statistical Analysis

Statistical analyses were performed using the SPSS software ver. 19.0, (IBM, Armonk, NY, USA). Categorical and dichotomous variables were expressed as percentages, and continuous variables as medians. The demographic and clinical data were compared between patients with and without PU on admission using Student’s *t*-test (level of significance of the test 99%) for continuous variables and the chi-squared (χ^2^) test for categorical and dichotomous variables. Normal distribution of variables was assessed by the Kolmogorov–Smirnov test. Variables found to be significantly different between the two groups were included as independent variables in a logistic regression model. Logistic regression was used to examine the association of different demographical data, admission diagnosis, and clinical features with PUs. Odds ratios (OR) and 95% confidence intervals (CI) were calculated. A univariate analysis was conducted to evaluate the association between PUs and age and sex. In all analyses, a *p*-value < 0.05 was considered significant.

## 3. Results 

### 3.1. Patient Characteristics

We analyzed the clinical data of 878 patients hospitalized in the Neurorehabilitation Unit of the University Hospital “G. Martino” of Messina over a period of seven years. According to the inclusion/exclusion criteria, 816 patients were enrolled in the study (479 males, average age 62 ± 16.56, range 15–96 years). Patients hospitalized for less than one week were excluded (n.62). The patients’ clinical characteristics and demographics are shown in Table 1.

### 3.2. Department of Origin, Initial Diagnosis, and Clinical Status

Prior to admission to the rehabilitation unit, patients were hospitalized in the following departments: neurology (n.409 = 50.12%), neurosurgery (n.191 = 23.40%), internal medicine (n.36 = 4.41%), ICU (n.30 = 3.67%), general surgery (n.21 = 2.57%), home care (n.128 = 15.68%), and nursing home (n.1 = 0.12%). General surgery units had the highest percentage of patients with PUs (47.62%), followed by ICU (17.82%), internal medicine (12.84%), neurology (14.18%), and neurosurgery (19.90%). The incidence of PU was very low (2.34%) in patients coming from home care settings. All reported data are statistically significant (χ^2^ = 26.594226, levels of freedom 4, *p* = 0.000024).

Among the patients admitted to the rehabilitation unit, vascular (stroke/SCI), traumatic brain injuries (TBI), and neoplastic diseases were the most common (Table 2). Vascular disorders were common in both sexes, with a female prevalence (χ^2^ = 11.46, *p* = 0.0007, OR 0.61). The most common clinical features associated with PU were paraplegia (62.5%), hemiplegia (25%), hemiplegia with aphasia (25.81%), tetraparesis (23.58%), hemiparesis (14.04%), hemiparesis with aphasia (14.03%), and paraparesis (7.64%). There was no sex difference between patients with or without PUs.

### 3.3. Localization of PUs

The most common locations of PUs were the heels (n.86 = 36.44%), sacrum (n.68 = 28.81%), and buttocks (n.33 = 13.98%). Less common sites were the lateral malleolus (n.11 = 4.66%), dorsum of foot (n.9 = 3.81%), calves (n.4 = 1.69%), trochanteric region (n.3 = 1.27%), scapula (n.3 = 1.27%), occipital region (n.2 = 0.84%), and other sites (n.15 = 6.36%). There is no clear association between the stage of the PU and its location. The worst PU grades (III, IV, and eschar) were mainly on the sacrum (n.20 stage III; n.8 stage IV) and heels (n.11 stage III; n.9 eschar). Among stage-III PUs (n.38), 53% were on the sacrum and 29% on the heel. All stage IV-PUs were on the sacrum and 75% were on the heel.

### 3.4. Risk Factors for the Development of PU

In univariate analysis, a statistically significant positive association of PU was found with (i) SCI-traumatic (OR 3.96), (ii) age ≥ 75 years (OR 2.09), and (iii) malnutrition (OR 3.91) (Table 3).

Among the patients studied, 371 (45%) were malnourished, with an increasing proportion in patients (95/131 = 73%) with a serum albumin level < 3.5 mg/dL on admission. In contrast, among the patients without PUs, 279 (41%) were malnourished (χ^2^ = 44.76; *p* = 0.0000, odd ratio (OR) 3.84). The incidence of PU in patients with serum albumin < 3.5 mg/dL was 25.53% (95/372), whereas in the group with albumin ≥ 3.5 mg/dL the incidence of PU was 8.10% (36/444). This difference is significant by chi-squared test (χ^2^ = *p* = 0.0000, OR 3.89).

### 3.5. Prevalence, Incidence, and Stage of PUs

A total of 131 patients (16.05%) presented with at least one PU on admission to the rehabilitation unit, with a total of 236 PUs (mean per patient 1.80 ± 1.07), a prevalence consistent with the literature [43,44,45]. 

No patients developed new PUs during the rehabilitation stay (incidence = 0). Age was a statistically significant parameter, as patients with PUs were significantly (*p* < 0.01) older than patients without PUs (67.05 vs. 61.05), with a minimum age of onset of 17 years and a maximum age of 88 years. A large proportion of patients (n.81 = 62%) with PUs were in the >65 age group (χ^2^ = 11.57, *p* = 0.003). Approximately half of the patients with PUs (n.68 = 51.90%) had a single lesion, 38 (29%) had two, and 25 (19%) had more than two (3–6) PUs. The most common stages of PU were stage II (n.118 = 50%) and stage I (n.60 = 25%). Stages III–IV accounted for almost 20% (III stage: n.38 = 16%; IV stage: 8 = 4%), and only 12 eschars were identified (5%). 

### 3.6. Risk Assessment Scales

The Norton and Braden scales (cut-off ≥ 16) had a sensitivity of 96.95%; the Waterlow scale (cut-off ≥ 17) had a sensitivity of 83.97%, but if the cut-off is ≥10, the sensitivity increases to 100%. SCIPUS (cut off ≥ 6) had a sensitivity of 96.67%. 

### 3.7. Development of Lesions 

During hospitalization, 178 out of 268 PUs (75%) were completely cured, especially those in stage I and partially those in stage II (116/118). A total of 35 PUs (15%) improved but were not completely cured (2 stage-II; 28 stage-III; 5 stage-IV), while 11 PUs (5%) remained unchanged (10 stage-III; 3 stage-IV) with a lower PUSH score at discharge compared to admission.

### 3.8. Length of Hospitalization

In our cohort, the mean time of hospitalization was 38.49 ± 29.28 days, with a significant difference in LOS between patients with (50.61 ± 40.52) and without (36.17 ± 26.01) PUs (*p* < 0.01 at T-Student test).

### 3.9. Rehabilitation Outcomes

On admission, patients with PUs had greater disability than those without PUs, as confirmed by both BI and FIM scores. The discharge scales showed lower scores in pressure ulcer patients compared to those without PU. Efficiency was also higher in patients without PUs, both for BI (0.62 vs. 0.41) and FIM (0.47 vs. 0.36), with a statistically significant result for BI (*p* = 0.0028) but no difference for FIM (*p* = 0.0712). There was a significantly higher efficacy in patients without PUs for both BI (34 vs. 20; *p* = 0.0000) and FIM (29 vs. 19; *p* = 0.0005).

## 4. Discussion

A PU is considered as any lesion resulting from unrelieved pressure that causes damage to the underlying tissue, and it is known to be a clinical challenge for both the physician and the patient [46]. The variable nature of an individual patient’s recovery response is related to many local and systemic factors, such as bacterial burden and infection, oedema, local pressure, wetness, chronic diseases or comorbidities (e.g., anemia, diabetes mellitus, renal or hepatic impairment), oxygenation, and the nutritional status of the tissue [47]. Therefore, PU management is often not immediate and can evolve in unpredictable ways, requiring a multidisciplinary approach [48]. Such data are confirmed by our cohort of patients, confirming that PUs are a major burden on the public health system, thus requiring valid preventive strategies. However, despite the growing interest in prevention, PUs remain a serious problem for patients, nurses, and physicians, as highlighted by several incidence and prevalence studies [49,50,51]. Ousey and colleagues supported the idea that PUs are an unpleasant complication of illness or disability [52]. Other authors suggested that PUs are iatrogenic damages and should be viewed as a negative quality care indicator [53,54]. In neurorehabilitation units, clinicians usually deal with patients with restricted mobility or impaired pain sensitivity which may predispose to PUs [55,56]. Proper management of PUs requires a multidisciplinary approach involving nurses, doctors, physiotherapists, occupational therapists, and other relevant healthcare professionals such as plastic surgeons [57]. To ensure effective management and reduce the incidence of complications such as sepsis or further wound development, this approach is essential [58]. Although a PU does not always progress according to the defined stages, if appropriate measures are not promptly taken at the injury onset, it can lead to ulceration [16]. It is, therefore, very important to detect PUs at an early stage, since they may heal more quickly than those ones at a later stage [36]. In fact, early detection and early consultation with such a specialized team may help to avoid PU worsening [57]. Late-stage PUs may require surgical intervention, such as debridement or reconstruction, which is not always possible due to significant morbidity, resulting in prolonged care [28]. The sacrum is the main site of PU development [59,60], although, in our sample, the heel was the most frequent site, probably because we considered sacral and gluteal regions as anatomically distinct locations. Consistently with other studies, a careful surveillance and assistance with appropriate and effective interventions allowed the risk of PU appearance during hospitalization in the rehabilitation departments to be eliminated [61,62,63]. Fortunately, in our cohort of patients, especially those with major brain damage, hospitalization in rehabilitation units cured and/or prevented PUs (mainly belonging to stages I-II) and other complications associated with reduced mobility like contractures. These results were achieved thanks to routinely applied activities such as exercise, bathing, and mobilization, combined with proper nutrition. Such skin prevention programs should be applied from the early stages of admission to intensive care units [64]. Another important issue for all collaborative patients is the education provided by the multidisciplinary team, providing adequate information on the measures patients can take to reduce their risk of developing pressure ulcers, e.g., the importance of maintaining a regular hydration level, good skin care, and regular fluid intake [65]. Our results confirm the relevance of this issue, also considering the increase in the ageing population and chronic-disabling diseases [66,67,68,69]. To limit the consequences of all preventable conditions, it is imperative to consider and limit the pathogenesis and risk factors in acute/post-acute patients [29]. Among the common risk factors for PUs (e.g., age, diabetes, vascular disease, and prolonged bed rest), malnutrition plays an important role according to our data, being present in 20–50% of new admissions and worsening in most cases during hospitalization [70], as confirmed by our data. Thus, it would be useful to offer a nutritional status screening at the beginning of hospitalization to prevent secondary complications [71]. Maintaining good nutritional status starts with adequate oral intake, and if this becomes inadequate or inconvenient, enteral or parenteral nutrition should be considered [20,72]. The aim is to achieve a positive nitrogen balance (approximately 30–35 calories/kg/day and 1.25–1.5 g protein/kg/day) by supplementing with vitamins and micronutrients, although supporting data are inconsistent [71].

The treatment of PUs includes regular repositioning of the patient, use of anti-bedsore mattresses to reduce or relieve pressure, and use of dressings to help heal the ulcer (i.e., alginate, hydrocolloid, foams, films, hydro fibers/gelling fibers, gels, and antimicrobial dressings) [48]. Then, if all these approaches have been proven to be inadequate because of the advanced stage of PUs, negative pressure wound therapy (NPWT) may be helpful, as suggested by its widespread acceptance in recent years [73]. The common indications for NPWT therapy are: chronic, acute, or traumatic injuries, partial- and full-thickness burns, dehiscence, diabetic ulcers, pressure wounds, and tissue flaps and grafts, particularly where increased fluid output is expected [74]. Normal PU healing is a process that includes many steps and proceeds from hemostasis to inflammation, granulation tissue production, re-epithelialization, and scarring [23]. NPWT achieves closed wound recovery, decreases oedema, increases perfusion, and clears infectious products and chronic inflammatory cells from the wound area [75,76]. The homogeneous negative pressure induces tissue and cell deformation, resulting in metabolic activity, fibroblast migration, and cellular growth, and also promotes the blood perfusion of the wound bed, leading in the release of leukocytes and plasma to counteract the chronic wound site [74]. However, in our cohort no patients required a NPWT. 

Finally, we outline the limitations associated with this type of study design. The data present problems due to the examination of medical records which were not originally created to collect patient information. The large volume of paper records could lead to the loss of patients’ information and potential bias. Then, there is the lack of a comparison group.

## 5. Conclusions

In conclusion, although PUs represent an economic burden for the healthcare system [77], due to their negative impact on treatment outcomes [78], delayed recovery [13], and prolonged hospital stay, an appropriate multidisciplinary approach may help to overcome such age-old problems. According to our results, the presence of PUs is linked to specific diseases (i.e., SCI-T), age (>75 years), and malnutrition. Furthermore, PUs seem to have a negative impact on the functional recovery of patients, as shown by the outcome scales and the average LOS. Particularly in neurorehabilitation units, PUs are a widespread problem that must be properly addressed by a multidisciplinary team of experts able to: (i) provide education and awareness of PU risk factors and preventive measures; (ii) implement patient risk assessment protocols to identify patients with an increased risk of developing PUs; and (iii) ensure that any treatments provided are effective and consider all possible risk factors for developing PUs.

## Figures and Tables

**Table 1:** Clinical and demographic characteristics of all patients.

	PUs	No PUs	*p*-Value
Total, n. (%)	131 (16.05)	685 (83.95)	
Sex			0.1038
Male, n. (%)	68 (51.91)	411 (60)	
Female, n. (%)	63 (48.09)	274 (40)	
Age (y)			
Total, mean (SD)	67.05 (15.12)	61.05 (16.66)	0.0001
Male, mean (SD)	63.68 (32.68)	58.7 (16.95)	0.0557
Female, mean (SD)	70.70 (13.78)	64.08 (15.71)	0.0022
Length of stay (d)			0.0000
Mean (SD)	50.61 (40.52)	36.17 (26.01)	
Albumin (mg/dL)			
Mean (SD)	3.17 (0.47)	3.52 (0.45)	0.0000
Disability (on admission)			0.0000
BI ≤ 45	126	422	
BI > 45	5	263	

BI: Barthel Index; d: days; SD: standard deviation; PU: pressure ulcer; y: years.

**Table 2:** Etiology of the disease of patients admitted to the neurorehabilitation unit.

No PU Patients				PU Patients			
Etiology	n. (%)	Male (%)	Female (%)	Etiology	n. (%)	Male (%)	Female (%)
Vascular	361 (53)	195 (47)	166 (61)	Vascular	73 (56)	36 (53)	37 (59)
Ischemic	250	126	124	Ischemic	42	17	25
Hemorrhagic	93	59	34	Hemorrhagic	25	14	11
Spinal cord	18	10	8	Spinal cord	6	5	1
Traumatic	60 (9)	48 (12)	7 (3)	Traumatic	20 (15)	14 (21)	6 (10)
TBI	36	29	7	TBI	6	4	2
SCI-T	17	12	5	SCI-T	12	9	3
TBI + SCI-T	7	7	0	TBI + SCI-T	2	1	1
Neoplastic	66 (10)	34 (8)	32 (12)	Neoplastic	14 (11)	4 (6)	10 (16)
Intracranial	51	23	28	Intracranial	9	4	5
Medullar	29	15	14	Medullar	5	0	5
MS	75 (9)	46 (10)	29 (9)	MS	0	0	0
Inflammatory	58 (8)	44 (11)	14 (5)	Inflammatory	13 (10)	10 (15)	3 (5)
Encephalitis	2	1	1	Encephalitis	1	0	1
Encephalomyelitis	14	11	3	Encephalomyelitis	6	5	1
Myelitis	24	18	6	Myelitis	3	2	1
Polyneuropathy	18	14	4	Polyneuropathy	3	3	0
Degenerative	45 (7)	32 (8)	13 (5)	Degenerative	3 (2)	3 (4)	0
Others	20 (3)	12 (3)	8 (3)	Others	8 (6)	1 (1)	7 (11)
Total	682	411	274	Total	131	68	63

MS: multiple sclerosis; PU: pressure ulcer; SCI: spinal cord injury; SCI-T: traumatic spinal cord injury; TBI: traumatic brain injury.

**Table 3:** Risk factor analysis.

	OR	*p*-Value	95% CI
Age (≥75 vs. <75)	2.09	0.0002	1.41–3.11
SCI-T	3.96	0.0002	1.85–8.51
Malnutrition vs. normal nutrition	3.91	0.0000	2.59–5.91
Traumatic vs. non traumatic injuries	1.96	0.146	1.13–3.39
Stroke	1.04	0.8221	0.72–1.52
Non traumatic SCI	0.86	0.5431	0.53–1.40
TBI	0.87	0.7486	0.36–2.10
Sex (females vs. males)	1.39	0.0848	0.95–2.02

CI: confidence interval; OR: odds ratio; SCI: spinal cord injury; SCI-T: traumatic spinal cord injury; TBI: traumatic brain injury.

## Data Availability

All data analyzed in this study are included in this published article.
